# Comparative Analysis of Fasting Blood Glucose and Salivary Electrolytes Concentrations among Individuals with Type II Diabetes: A Randomized Controlled Hospital Based Study^☆^

**DOI:** 10.1016/j.toxrep.2022.05.022

**Published:** 2022-06-03

**Authors:** Victor Onyemaechi Egboh, Peggy Ejiro Ohwin, Tarela Melish Elias Daubry, Ofioritse Ogheneyoma Ofulue, Bartholomew Chukwuebuka Nwogueze, Evelyn Tarela Ojugbeli, Uchechukwu Levi Osuagwu, Eze Kingsley Nwangwa

**Affiliations:** a Department of Human Physiology, Delta State University, Abraka, Nigeria; b Department of Medical Biochemistry, Delta State University, Abraka, Nigeria; c Translational Health Research Institute (THRI), School of Medicine, Western Sydney University, Campbelltown, NSW 2560 Australia

**Keywords:** Diabetes mellitus, Salivary electrolytes, Fasting blood glucose, Nigeria, Type 2 diabetes

## Abstract

Salivary gland dysfunction is common in people with diabetes. This study aimed to compare the measurements of salivary electrolytes (SE); Na^+^, K^+^, Cl^-^ and HCO_3_^-^ between diabetes and an age matched control group, and assess the relationship between fasting blood glucose (FBG) and salivary electrolytes, and salivary glucose (SG). Eighty-five human participants [diabetes group, n = 45 (23 males and 22 females) and control group, n = 40 (20 males and 20 females)] aged between 25 and 65years were tested. Saliva samples were taken between 7.00 am and 8.00 am after an overnight fast and SG and SE concentrations were analysed**.** Diabetes mellitus was defined using FBG ≥ 126 mg/dl. SG and SE concentrations were analysed using *t*-test and Pearson Correlation Coefficient tested the relationship between FBG and Salivary electrolytes and glucose. The participants were matched in their baseline demographic characteristics with a mean age of 49 years (standard deviation SD, 11 years), body mass index (25.7 kg/m^2^ (SD, 3.6). Half of them were males (50.6 %) and predominantly traders (30.6 %). However, the mean values for the salivary sodium, potassium, chloride and bicarbonate electrolytes were significantly higher in the diabetes group compared with the control group (P < 0.05). Of the salivary electrolytes, only the bicarbonate was significantly correlated with FBG (*r* = −0.594, p = 0.004) in female participants. This study found that people with diabetes have elevated salivary electrolytes which were not dependent on their age and gender. Although this study suggests some potential for saliva as an alternative in monitoring of diabetes mellitus, extensive research is required before we can reach any firm conclusion.

## Highlights

• Electrolytes in diabetic is elevated in salivary glucose compared to non-diabetic.
• Elevated salivary electrolytes in Diabetes are not dependent on age and gender.
• Salivary electrolytes in diabetic patient are gender specific..
• Saliva samples are potential diagnostic marker used in monitoring diabetic patients.

## Graphical Abstract

**Figure:**
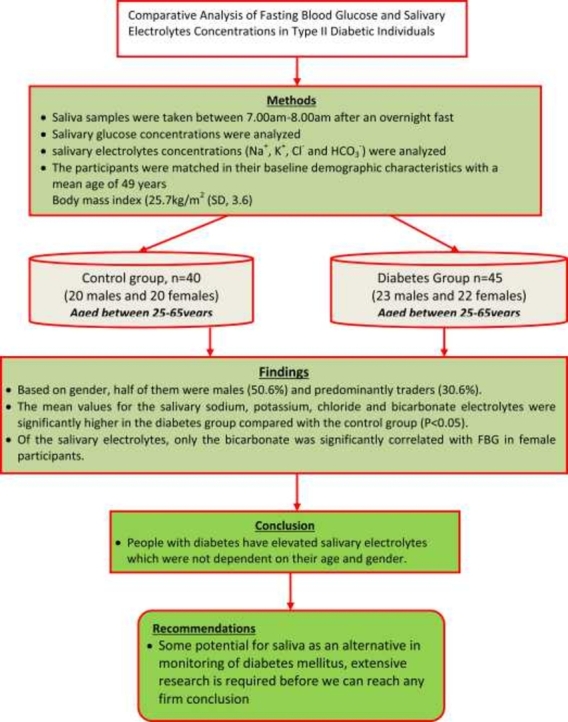

## 1 Introduction

Diabetes mellitus (DM) is a group of metabolic disorders of carbohydrate metabolism in which glucose is underutilized, producing hyperglycaemia. It is characterized by relative or absolute insufficiency of insulin secretion and/or concomitant resistance to the metabolic action of insulin on target tissues [1]. Diabetes mellitus is a major public health problem globally as the prevalence and burden are uncontrolled in urban areas due to significant lifestyle choices and it has been described as an epidemic [1], [2], [3]. Globally, it was reported in 2016 that more than 422 million adults were living with diabetes and the prevalence is expected to increase by 55 % to over 591.9 million by 2035 [4]. Three-quarters of those with diabetes live in Low to Middle Income Countries (LMIC) such as Nigeria, and this is projected to increase [5]. Using figures from population-based studies from 12 countries in SSA, Manne-Goehler et al. [6] puts the median prevalence level of DM in sub-Saharan Africa at 5 %. In Nigeria, recent reports showed an incidence of diabetes of 2.2 % and 90 % have type-2 diabetes mellitus [3].

Saliva research is rapidly advancing because of the use of novel approaches such as metabolomics, genomics, proteomics and bioinformatics [7]. Saliva is an exocrine secretion of the salivary glands that contains mostly water (99 %), electrolytes, proteins, and enzyme [8]. It is often referred to as the “mirror of the body” as it is the indicator of health not just in the oral cavity but also throughout the body. Saliva plays an important role in maintaining the equilibrium of the oral ecosystem by providing the sensory perception of food, aids in chewing, swallowing, and food digestion [8]. Whole saliva contains locally produced as well as serum-derived biomarkers that have been found to be useful in the diagnosis of a variety of systemic disorders such as Grave’s diseases, Rheumatoid arthritis, hypertension, Myasthenia gravis. Understanding the role of each salivary component in the oral cavity homeostasis is crucial to perceive how its changes or absence may be linked with pathological conditions.

Multiple epidemiologic studies have suggested that diabetes is a risk factor for the development of oral diseases in humans [5]. Diabetes besides damaging various systems of body may also impair salivary gland functions, which leads to a reduction in the salivary flow and changes in saliva’s composition and Periodontitis [9], [10]. Screening for diabetes in general practice by measuring fasting blood glucose levels is feasible, stressful, invasive and time consuming. Different strategies have been suggested to improve diabetes detection and there has been increased interest towards non-invasive method to diagnose this disease, including the use of the saliva sample [11]. Salivary gland hypofunction has also been reported to be frequent in people with diabetes majorly due to overall diminished flow of saliva which is a consequence of dehydration which might contribute to their susceptibility to oral infections like candidiasis, dental caries, xerostomia, etc [12].

There is paucity of information on the relationship between salivary electrolyte and glucose levels and fasting blood glucose in people with type-2 diabetes. In a 2019 study conducted in Bayelsa State, Nigeria, the authors included 100 participants (50 each, with and without diabetes) but failed to segregate between diabetes types even though the measured parameters vary significantly with diabetes type [12], [13]. They found that the biochemical parameters of the blood and saliva were comparable between people with and without diabetes though not correlated. However, the recruitment of the participants into treatment and control arm was not randomized, exposing the study to various bias that may have affected the reliability of their results [14], [15]*.* Therefore, the aim of this studywas to determine the relationship between fasting blood glucose (FBG) and both salivary electrolytes, SE (Na^+^, K^+^, Cl^-^ and HCO_3_^-^) and salivary glucose in people with Types 2 diabetes. These were compared with an age matched controlled group.

## 2 Materials and methods

### 2.1 Study setting, design and recruitment

This research is a randomized cross-sectional, analytical study involving 85 participants with diabetes of ≥ 2 year’s duration attending the Endocrine Unit of the Medical out-patients Department, Central Hospital, Agbor, Delta State, Nigeria. The age of the participants ranged from 25 to 65 years with a Fasting blood glucose (FBG) concentration of 126 mg/dL (7 mmol/L) or higher as at the time of sample collection.

Sample size was determined according to Oshilonya et al. [3]. A total of eighty-five (85) participants was determined as adequate to detect significant differences at 95 % confidence intervals. Eligible participants who attended the clinic between January 22nd 2018 and March 20th 2018 were invited to participate in this study. Stratified random sampling was employed to recruit subjects for this study. Randomization and questionnaire administration was performed by a Pharmacist intern who was not involved in the study. Randomization took place between 8 and 10th February, 2018. Subjects were divided into male and female strata/groups. Male stratum consisted of 45 subjects for test group and 42 subjects for control. Each strata/group was subjected to simple random sampling. Participants were selected through balloting with Yes or No options. Those who selected “Yes” and fulfilled the inclusion criteria for the research were selected. Twenty-three (23) male subjects between 25 and 65 years were selected for test group while 20 was selected for control. Twenty-two (22) female subjects were selected for test group and 20 for control.

### 2.2 Inclusion and exclusion criteria

For this study, only people who have had type 2 diabetes for 2 or more years duration were included. The following participants were excluded: pregnant women, participants with tumor of the salivary gland, Sjogren’s syndrome, xerostomia, those with a past history of salivary gland surgeries, those receiving radiotherapy around head and neck region, and people who were taking other non-diabetes medications and those with severe diabetes related complications were excluded. The study lasted for two months (January 22nd to March 20th 2018).

### 2.3 Sample Collection

A research team member (OEP) blinded to the treatment group collected the participants’ saliva samples. The study adopted the use of salivary glucose measurements to detect diabetes [16]. Two millilitres (2 ml) of saliva samples were taken between 7.00 am and 8.00 am after an overnight fast. Participants were asked to spit (after rinsing their mouths with deionized water) into plastic vials [13]. Samples were centrifuged at 6000 rpm for 10 min. The supernatant obtained were stored at 4 °C and subsequently analysed within six hours. For collection of blood samples, two milliliters of blood was collected intravenously and the sample was transferred into a fluoride oxalate sample container. Both methods are standardized methods of sample collection approved by World Health Organization (WHO) as stated previously [12]. The same assessor collected samples from all participants in both groups using the same protocol.

### 2.4 Analysis of the collected samples

Another assessor (EVO) analysed all samples in this study using standardized methods.

#### 2.4.1 Glucose oxidase end-point method

Salivary glucose and FBG estimation was performed using the glucose oxidase end-point method described previously [17]. For this, 1000 micro1iters of reagent solution was pipetted into three test tubes labeled ‘Blank,' ‘Standard,' and ‘Test’. In the ‘Standard' test tube, 10 microliters of standard was put, followed by 10 micro1iters of test sample in the ‘Test' test tube. Before aspiration, all of the test tubes were thoroughly mixed and incubated using an incubator. This was stored at a temperature of 37 °C for ten minutes. The analyzer was first filled with a reagent blank, followed by a standard solution, and the reading was obtained. Finally, it was filled with the test sample and the reading was again taken. The results were computed, and the milligrams per deciliter (mg/dl) values were used.

#### 2.4.2 Flame atomic emission spectrophotometric method

The serum electrolytes Sodium and Potassium was performed using flame emission photometrymethod, which was based on the desolvation of a solution containing these elements by a flame, leaving a solid (salts) that dissociates to neutral ground state atoms [18]. These atoms become excited in the flame, shifting to a higher energy state, and then returning to the ground state, emitting light of a specific wavelength (589 nm for sodium and 768 nm for potassium). The quantity of current created is proportional to the amount of sodium or potassium contained in the original sample after the light passes through an appropriate filter onto a photosensitive element.

#### 2.4.3 Back titration method

The serum level of bicarbonatewas determined according to Back titration method [19]. The method is based on the release of carbon dioxide from the bicarbonate ion in serum when dilute hydrochloric acid is added. With phenol red as an indicator, the excess acid was titrated with sodium hydroxide.

#### 2.4.4 Titration method

The serum level of chloride (Cl) was determined according to titration method [20]. A standardized mercuric nitrate solution was used to titrate the sample in the presence of diphenylcarbazone as an indicator. Mercury chloride (HgCl) is formed when chloride ions (Cl^–^) in the sample combine with mercuric ions (Hg^2+^) to form a colorless, soluble, but only slightly ionized compound. In the presence of diphenylcarbazone, the excess mercuric ions generate a violet tint when all chloride ions have been complexed. Test tubes were labeled blank, standards and samples. Chloride reagent (4.5 cm^3^) was pippeted into each test tube and 30 microliters of standards or samples were added to respective tubes, mixed and incubated at room temperature for five minutes. The spectrophotometer was set to 480 nm and zero with reagent blank. The absorbance readings of all the tubes were obtained and recorded.

### 2.5 Ethics and consent

Ethical approval for this study was obtained from the Research and Bioethics committee of the Faculty of Basic Medical Sciences, Delta State University, Abraka (Ref no: RBC/FBMS/DELSU/18/01) as well as from the Ethics Committee of the Central Hospital, Agbor, Delta State, Nigeria. The study protocol adhered to the tenets of the Declaration of Helsinki. Prior to recruitment into the study, a written consent was sought and obtained from all participants after a detailed explanation of the study protocol has been provided to them.

### 2.6 Statistical analysis

Descriptive statistics (mean values ± standard deviation (SD), range, proportions and percentages) were used to express the concentrations of analytes. Within group analysis to compare male versus female measurements was conducted using student *t*-test while one way Analysis of variance (ANOVA) was used to compare between groups (diabetes versus control). Pearson correlation (*r*) was used to assess the relationship between fasting blood glucose and salivary electrolytes and salivary glucose. A *p*-value of < 0.05 was considered statistically significant.

## 3 Results

### 3.1 Demographic characteristics of the study participants

Table 1 shows the demography of the study participants as well as the measurements of the salivary electrolytes. Of the eighty-five participants in this study, there were 45 in the diabetes group (52.9 %) and 40 in the control group (47.1 %). The participants were matched in their baseline demographic characteristics with a mean age of 49 years (standard deviation, 11) years), body mass index (25.7 kg/m^2^ (SD, 3.6). Half of them were males (50.6 %) and predominantly traders (30.6 %). However, the mean values for the salivary sodium, potassium, chloride and bicarbonate electrolytes were significantly higher in the diabetes group compared with the control group (Table 1).

**Table 1:** Demographic characteristics and the measured variables in diabetes and control participants.

*Variables*	*Total (n = 85)*	*Control (n = 40)*	*Diabetes (n = 45)*	*p value*
Demography				
Age, mean (SD); range	49 (11); 26–71	50 (12); 30–71	48 (11); 26–65	*0.251*
Gender, n (%)				
*Male*	43 (50.6)	20 (50.0)	23 (51.1)	*0.919*
*Females*	42 (49.4)	20 (50.0)	22 (48.9)	
Occupations, n (%)				
*Teaching*	7 (8.2)	3 (7.5)	4 (8.9)	*0.922*
*Civil service*	19 (22.4)	9 (22.5)	10 (22.2)	
*Trading*	26 (30.6)	13 (32.5)	13 (28.9)	
*Farming*	19 (22.4)	9 (22.5)	10 (22.2)	
*Others*	14 (16.5)	6 (15.0)	8 (17.8)	
Anthropometry, mean (SD)				
*Weight, kg*	80.68 (7.09)	82.25 (6.80)	79.29 (7.13)	*0.054*
*Height, m*	1.78 (0.10)	1.80 (0.10)	1.76(0.09)	*0.071*
*BMI, kg/m2*	25.7(3.6)	25.6(3.7)	25.8(3.7)	*0.866*
Salivary samples				
*Salivary Glucose, mg/dl*	10.34(7.71)	11.15 (7.88)	19.63 (7.58)	*0.369*
*Salivary Sodium (Na*^*+*^*)*	25.80 (15.48)	17.95(9.57)	32.77 (16.45)	*< 0.0005*
*Salivary Potassium (K*^*+*^*),*	23.68 (15.58)	13.81(4.99)	32.45 (16.55)	*< 0.0006*
*Salivary Chloride (Cl*^*-*^*)*	16.59(13.6)	9.05(5.33)	23.30 (15.19)	*< 0.0007*
*Salivary Bicarbonate (HCO*_*3*_^*-*^*)*	6.43(4.89)	3.99 (2.96)	8.60(5.25)	*< 0.0008*

Values are expressed as mean ± standard deviations of their standard units of measurement.

P values are one way ANOVA results between diabetes and control group. P < 0.05 is significant.

Salivary electrolytes are in mmol/L.

BMI = Body mass index.

### 3.2 Correlations of fasting blood glucose and salivary electrolytes in male and female participants

The mean FBG in this study was 180.4 (SD, 54.5) in the diabetes group. Table 2 presents the results of Pearson correlation coefficient between FBG and salivary electrolytes in the diabetes group. The mean FBG measurements showed similar positive correlations with salivary glucose and similar negative correlations with the salivary electrolytes (sodium, chloride, potassium and bicarbonate) electrolytes in both male and female participants. However, there was a significant relationship between FBG and salivary electrolytes for the female participants (*r* = −0.594, p = 0.0004) but not for the males.

**Table 2:** Correlations (r) of mean salivary glucose and salivary electrolytes of male and female participants with diabetes.

Gender Diabetic grouping	FBG	Salivary glucose	Salivary Na^+^	Salivary K^+^	Salivary Cl^-^	Salivary HCO_3_^-^
Males (n = 23)	166.65 (40.68)	8.31 (7.60)	30.96 (18.43)	31.65 (18.18)	23.08 (16.70)	8.18 (6.10)
R	constant	0.316	-0.124	-0.036	-0.03	-0.018
*p-value*		*0.142*	*0.574*	*0.871*	*0.891*	*0.936*
Females (n = 22)	194.73 (63.81)	11.01 (7.48)	34.66 (14.28)	33.28 (15.05)	23.52 (13.82)	9.05 (4.30)
R	constant	0.121	-0.119	-0.227	-0.384	-0.594
*p-value*		*0.591*	*0.599*	*0.309*	*0.077*	***0.004***

Values are expressed as mean ± standard deviations of their standard units of measurement.

Pearson Product Moment Correlation Coefficient. Bolded are significant p values (p < 0.05).

FBG= Fasting blood glucose.

### 3.3 Gender based differences in salivary glucose and electrolytes of people with diabetes

Table 3 shows the result of student *t*-test comparisons of the mean FBG and salivary electrolytes between male and female participants in the diabetes and the control groups. Although the mean values of the salivary electrolytes were higher in males than females for the control group, and lower in males than females in the diabetes group, the differences did not reach statistical significance in both groups (p > 0.05, for all comparisons).

**Table 3:** Comparison between male and female participants’ glucose and salivary electrolyte measurements in both group.

Electrolyte variables	Controls			Diabetes		
	Male	Female	*p*-value	Male	Female	*p*-value
Salivary Glucose (mg/dl)	7.78 (6.40)	7.64 (4.97)	0.94	8.31 (7.59)	11.01 (7.48)	0.24
Salivary Sodium (Na^+^)	18.71(10.67)	17.20(8.54)	0.62	30.96(18.42)	34.66(14.28)	0.46
Salivary Potassium (K^+^)	14.34(5.42)	13.28(4.59)	0.51	31.65(18.18)	33.28(15.05)	0.74
Salivary Chloride (Cl^-^)	9.58(5.87)	8.53(4.83)	0.54	23.08(16.70)	23.52(13.82)	0.92
Salivary Bicarbonate (HCO_3_^-^)	3.70(3.13)	4.28(2.84)	0.54	8.18(6.09)	9.05(4.30)	0.58
FBG (mg/dl)				166.65(40.68)	194.73(63.81)	0.09

Values are expressed as Mean (SD). Values that bear the superscript ‘a′ on a column differ significantly (p < 0.05).

## 4 Discussion

The findings of this control study revealed that people with diabetes had significantly higher mean values for salivary electrolytes (sodium, potassium, chloride and bicarbonate) compared with an age and gender matched control group. In people with diabetes the salivary electrolytes were twice as high as for those without diabetes which corroborated previous studies [21]. The salivary glucose level was higher in diabetic subjects when compared to non-diabetic subjects but the observed elevation was not significance. High salivary glucose in conjunction with diminished flow of saliva has also been reported to be responsible for the complaint of dry mouth and the oral mucosal lesions due to microvascular abnormalities observed in people with diabetes [22], [23].

With respect to potassium, salivary concentration of this ion was found to be increased in participants with diabetes when compared with those without diabetes. Similar finding has been reported by Mata et al., [24] and Lasisi and Fasanmade, [10]. Elevation of potassium concentration in saliva of participants with diabetes is probably secondary to diabetes and causes a decrease in salivary fluid output which can lead to hyperchloremia, one of the complications associated with diabetes characterized by an electrolyte imbalance that occurs due to too much chloride in the blood [25], [26].

This present study did not find a significant association between FBG and Salivary glucose and between FBG and Salivary electrolytes (Na^+^, K^+^, Cl^-^) in both male and female participants (Table 2). However, previous studies have reported lower flow rate and composition of the saliva in female participants which was attributed to the smaller salivary glands and hormonal pattern in females [27], [28]. Deshpande et al. [29] reports that potassium, chloride and calcium levels were higher in females compared to males. Some studies have however reported that total unstimulated salivary flow and composition were not influenced by gender and size of the salivary gland but stimulated salivary flow and composition was directly related to the size of the salivary gland which is consistent with findings in the present study [30], [31]. However, significant negative association between FBG and Salivary electrolyte (HCO_3_^-^) was observed in female diabetic subjects. The mechanism behind the increased level of salivary electrolytes and its relationship with fasting blood glucose (FBG) in our study is not fully understood.

Despite the elevated salivary glucose and salivary electrolytes observed among female diabetic subjects when compared to their male counterpart, no significant difference was observed in these parameters (Table 3). Due to the fact that diabetic subjects have salivary gland dysfunction which makes them prone to decreased mucosal integrity, most studies suggests that saliva cannot be used to indicate blood glucose in diabetics as the amount of membranous damage to the salivary gland in turn increases the quantity of leakage of glucose from plasma to saliva is unpredictable [24], [31], [32]. Twetman, et al. [22] justified the threshold mechanisms along the basement membrane; hence rejected the possibility of existence of any relationship between serum and saliva glucose. Other study results were in accordance with these findings [32], [33].

## 5 Conclusions

This study found that the sodium, potassium and bicarbonate electrolytes obtained in people with diabetes mellitus are significantly elevated with similar salivary glucose compared with an age and gender balanced group of people without diabetes. Fasting blood glucose and salivary bicarbonate electrolytes showed negative correlation in female participants with diabetes but not in the male participants, thus suggesting that saliva samples may be a potential diagnostic marker and could be used in monitoring people with diabetes. However, extensive research is required to confirm these findings and in different populations.

## CRediT authorship contribution statement

**Egboh, V.O** conceptualized and funded the study; **Ohwin, E.P** was involved in data curation and drafting of methodology; **Nwangwa E.K.** supervised the study and edited the manuscript; **Daubry, T.M.E** was in-charge of project administration; **Nwogueze BC** reviewed and edited the manuscript**; Uchechukwu,** L.O conducted biochemical investigation; **Ojugbeli E.T** searched for literatures; All authors read and approved the publication of the Manuscript.

## Limitation and strengths

Time constraint in sample collection as some of the patients do not present at the stipulated time. This was mitigated by making a reminder call to participants to enable them present on time. Team members also visited certain participants who could not afford the transport to collect the sample at the comfort of their homes. Another limitation is the uncertainty in fast duration. The participants of this study ought to observe an overnight fast as part of the requirement. However, it is not certain if they adhered strictly to this instruction.

## Declaration of Competing Interest

The authors declare that they have no known competing financial interests or personal relationships that could have appeared to influence the work reported in this paper.
